# Metabolic reprogramming, macrophage polarization, and intercellular crosstalk in pelvic inflammatory disease: emerging paradigms and therapeutic opportunities

**DOI:** 10.3389/fimmu.2026.1916689

**Published:** 2026-09-18

**Authors:** Shangtao Liu, Tongyue Liu, YongTing Li, Suo Zhang, Qiumei Dong

**Affiliations:** College of Traditional Chinese Medicine, Inner Mongolia Medical University, Hohhot, China

**Keywords:** inflammation, macrophage polarization, macrophages, metabolism, pelvic inflammatory disease

## Abstract

Pelvic inflammatory disease (PID) is a condition caused by the ascension of pathogenic microorganisms from the lower genital tract to the uterus and/or fallopian tubes, and is one of the most common clinical syndromes among women of reproductive age. The long-term sequelae of PID, including chronic pelvic pain, infertility, and recurrent episodes, significantly impair patients’ quality of life. However, the current clinical understanding of the immunopathological mechanism of PID remains insufficient, and there are no targeted therapeutic approaches. In recent years, macrophage polarization has been demonstrated to play a core regulatory role in the initiation, progression, and resolution of inflammation, and metabolic reprogramming is the key intrinsic mechanism driving the phenotypic transformation of macrophages, playing a crucial role in various diseases. At the same time, the bidirectional communication network between macrophages and endometrial cells has also been proven to play an important role in the disease progression. Therefore, clarifying the metabolic adaptation mechanism regulating macrophage polarization and its interaction with endometrial cells can provide potential targets for exploring new therapeutic strategies that target macrophage metabolism or block abnormal cell communication, which is crucial for developing targeted treatments that can restore immune homeostasis and alleviate the pathological damage of PID.

## 1 Introduction

Pelvic inflammatory disease (PID) is a condition caused by the ascension of pathogenic microorganisms from the lower genital tract to the uterus and/or fallopian tubes, encompassing endometritis, salpingitis, tubo-ovarian abscess, and pelvic peritonitis. It is one of the most common clinical syndromes among women of reproductive age. During the progression of PID, long-term sequelae such as chronic pelvic pain, infertility, and recurrent episodes are frequently observed, which severely affect patients’ quality of life (1–3). The majority of PID cases are associated with exogenous infections (such as Neisseria gonorrhoeae, and Chlamydia trachomatis), autoimmune diseases, and tissue damage (1, 3, 4). The key pathological features of PID include immune dysregulation, dysbiosis of the endometrial microbiota, abnormal expression of hormone receptors, and changes in endometrial contraction patterns driven by inflammation and fluctuations in hormone levels (1, 3, 5). Currently, PID affects 4% to 12% of women of childbearing age. Although PID rarely poses a threat to life, its long-term pathological effects are very significant, thus bringing a considerable disease burden. However, clinical efforts to prevent PID and to develop new treatment strategies for PID have made limited progress (6). Therefore, a deeper understanding of the immune-pathological mechanisms of PID, to regulate key cells and molecules that control the resolution of inflammation and tissue fibrosis, is of great significance for developing new treatment strategies and improving the long-term prognosis of patients.

Macrophages are among the most abundant immune cells in the pelvic microenvironment and play multiple roles in embryonic implantation and host defense (7, 8). Macrophages possess high plasticity and can polarize into functionally distinct phenotypes in response to different microenvironmental signals. Current research predominantly focuses on the dynamic process of macrophage polarization into M1-type macrophages (M1) and M2-type macrophages (M2).In the context of infection, M1-like macrophages can secrete pro-inflammatory cytokines to control infection, whereas M2-like macrophages predominantly secrete factors such as transforming growth factor to promote tissue repair and immune tolerance. However, excessive or sustained M1-like polarization can exacerbate tissue damage, while aberrant M2-like polarization can lead to tissue fibrosis and adhesions. Therefore, the functions of macrophage subpopulations with different polarization tendencies should be properly regulated (9).

In recent years, the role of macrophage polarization in autoimmune inflammatory diseases such as rheumatoid arthritis has been extensively studied (10). However, in the field of infectious diseases such as PID, the underlying mechanisms remain largely unexplored. Metabolic reprogramming has been shown to play a crucial role in regulating the M1/M2 balance, indicating that changes in the body’s metabolic state within the infected microenvironment can help alleviate or exacerbate the inflammatory response (11). Moreover, it is more complex: macrophages do not act independently but form a close, bidirectional communication network with endometrial cells. Macrophages in the inflammatory environment can alleviate or aggravate endometrial inflammation manifestations by polarizing or upregulating the expression levels of related proteins (such as HO-1), and conversely, damaged endometrial cells can recruit macrophages through autophagy reactions and the secretion of different functional cytokines to stimulate these cells to produce pro-inflammatory cytokines, forming a self-sustaining inflammatory cycle (12–14). Therefore, a deeper understanding of the impact of metabolic reprogramming on macrophage polarization in PID, as well as the communication between macrophages and endometrial cells, is of great significance for further exploring PID treatment strategies.

## 2 The role of macrophages in inflammation

### 2.1 The source of macrophages

Macrophages are phagocytic immune cells in innate immunity. There are two main sources of macrophages: one is derived from monocytes (monocyte-derived macrophages); the other originates from progenitor cells in the embryonic yolk sac and develops into self-renewing tissue-resident macrophages. As the sentinel of the immune system, macrophages can continuously monitor potential threats and respond quickly when needed. Macrophages from different sources are crucial components of human innate immunity (15, 16). Macrophages normally perform three basic functions: antigen presentation, immune regulation, and phagocytosis. In the human body, macrophages mostly show a high degree of plasticity and heterogeneity, and play a key role in a variety of biological activities, such as a range of homeostatic functions and adaptive immune responses to host defense, such as regulation of reactive oxygen species (ROS), maintenance of iron homeostasis, and supervision of metabolic functions (17, 18).

Recent studies have shown that macrophages are closely associated with many inflammatory immune diseases in humans, including malignant tumors, atherosclerosis, pancreatitis, and inflammatory bowel disease (19–22). Studies have shown that there is a complex interaction between cellular state in the inflammatory response and macrophage activation. Under an inflammatory environment, the cellular stress response can lead to macrophage activation through mitochondrial stress, autophagy, endoplasmic reticulum stress, hypoxic stress, and other mechanisms, thereby significantly affecting disease progression. In addition, substantial evidence indicates that abnormal macrophage expression is closely associated with the prolongation of chronic inflammation, and a high macrophage count is often associated with drug resistance, which can be improved by targeted regulation of macrophages (23–27). Therefore, given the importance of macrophages in inflammatory diseases, macrophage polarization has also become a focus of current research as a key mechanism by which they regulate inflammation.

### 2.2 Macrophage polarization

Macrophage polarization refers to a response mode in which macrophages are activated in a specific environment, primarily influenced by epigenetic and cell survival pathways, the tissue microenvironment, and extrinsic factors such as microbial products and cytokines released during inflammation (28).

In disease states, macrophages can be recruited from the bone marrow as monocytes to the inflamed and infected tissues. In addition, when abnormal signals occur in the human microenvironment, such as inflammatory responses and apoptosis, macrophages can express receptors that recognize these signals and polarize to M1 or M2 phenotypes, owing to their outstanding plasticity (29–31). In general, M1 exhibits pro-inflammatory functions with antibacterial and antitumor activities, while M2 exhibits anti-inflammatory functions and promotes tissue repair. Therefore, the normal expression of macrophage polarization function is essential for effectively delaying disease progression and promoting tissue repair. However, if this coordinated balance is disrupted, it may lead to adverse disease progression (32). Therefore, clarifying the functions of M1 and M2 and further exploring the dynamic mechanism of macrophage polarization in diseases are essential for clinical macrophage-targeted therapy.

M1 and M2 are currently the two most extensively studied phenotypes in macrophage polarization research. However, macrophage polarization is not a simple direct switch between cell types, but rather a continuous and complex dynamic process. For instance, prior to polarization, macrophages exist in a non-polarized state known as M0 (33, 34), Moreover, as research has progressed, scientists have identified additional phenotypes in the polarization process, such as M4 and Mox (35), as well as the intermediate phenotype M3 (36)., which represents a transitional state between M1 and M2. Therefore, M1 and M2 serve as simplified operational phenotypes within a conceptual framework, rather than fixed macrophage subsets. Polarization toward these two phenotypes is deeply involved in the pathogenesis of inflammatory, autoimmune, and metabolic diseases (37, 38). A deeper understanding of this dynamic polarization process will contribute to a more profound comprehension of the pathogenesis of various diseases.

During inflammation, macrophages shift between M1 and M2 phenotypes, and the proportions of these shifts change as the disease (39). When exposed to stimuli such as lipopolysaccharide (LPS), tumor necrosis factor-α (TNF-α), IL-12, and IL-23, resting macrophages tend to polarize toward the M1 phenotype (17), characterized by high expression of inducible nitric oxide synthase (iNOS), CD80, CD86, Toll-like receptor 4, and pro-inflammatory cytokines (e.g., IL-1β, TNF-α, IL-12), thereby exerting antimicrobial and antitumor activities. However, excessive M1-like polarization may also impede tissue healing and regeneration (17, 40–42). In contrast, IL-4 and IL-10 can induce macrophages to shift toward the M2-like phenotype (43, 44), characterized by upregulation of arginase-1 (Arg1), CD206, CD163, CD209, and anti-inflammatory cytokines (e.g., IL-10), which participate in Th2-type immune responses, parasite clearance, and inflammation suppression, helping to alleviate inflammation and promote tissue repair (17, 45–47). Based on activation patterns induced by different cytokines, macrophage polarization toward M2 can be further classified into four subtypes—M2a, M2b, M2c, and M2d—which are respectively regulated and induced by cytokines and receptors such as IL-4, Toll-like receptor (TLR) agonists, IL-10, and activated adenosine A2A receptors (48). However, in *in vivo* pathological environments, the boundaries between these subtypes are often blurred and dynamically changing, and their functional significance warrants further investigation.

It has been shown that macrophages use their pattern recognition receptors and produce highly reactive ROS and reactive nitrogen to enhance antimicrobial responses during bacterial and fungal infections (48–51). At the same time, influenced by autocrine IL-10, such as fibrin, in the tissue microenvironment, macrophages can accelerate their polarization toward the M2 anti-inflammatory phenotype to coordinate the inflammatory response (52, 53).

Therefore, macrophages have a high degree of plasticity, and the polarization response of macrophages is deeply involved in the process of human inflammatory infection and other diseases. When the dynamic balance of polarization is disrupted, it can exacerbate tissue and organ damage in the body (17). Therefore, in view of the important role of macrophage polarization in diseases, this paper further discusses the related factors affecting macrophage polarization, in order to provide reference for further research on targeting macrophages for disease therapy.

## 3 Factors affecting macrophage polarization

### 3.1 Regulation of signaling pathways on macrophages

Signaling pathways and transcription factors are critically important for regulating macrophage function, profoundly influencing their activation, polarization, and proliferation. The major ones involved in these processes include the nuclear factor κB (NF-κB) signaling pathway, the Janus kinase (JAK)/Signal Transducers and Activators of Transcription (STAT) signaling pathway, the Phosphatidylinositol 3-kinase (PI3K)/protein kinase B (Akt) signaling pathway, the c-Jun N-terminal kinase (JNK) signaling pathway, the CCAAT/enhancer-binding protein (C/EBP) family, the Notch signaling pathway, and others.

#### 3.1.1 NF-κB signaling pathway

NF-κB is a dimeric transcription factor that plays a critical role in inflammation and immunity by regulating the expression of various chemokines, cytokines, transcription factors, and regulatory proteins. NF-κB is expressed in almost all human cell types and controls the production of diverse pro-inflammatory mediators. Dysregulation of this pathway may contribute to the development of various autoimmune and inflammatory diseases (54, 55). NF-κB is the primary transcription factor in M1 macrophage polarization, and M1-like polarization can be induced by mechanosensitive pathways involving focal adhesion kinase and NF-κB (56). During infection, the NF-κB p65/p50 complex promotes the expression of pro-inflammatory cytokines such as TNF-α, IL-1β, and IL-6, thereby governing polarization and further amplifying inflammatory signals. This process is generally associated with Th1-related inflammatory immune responses (18).

#### 3.1.2 JAK/STAT signaling pathway

The JAK/STAT signaling pathway mediates a variety of cytokine-induced biological responses (57). The JAK/STAT pathway can be modulated by numerous intrinsic and environmental stimuli, thereby increasing the plasticity of cellular or tissue responses (18). Macrophage polarization is influenced by the JAK/STAT pathway, which has been validated *in vitro* and in diseases such as rheumatoid arthritis and inflammatory bowel disease (58, 59).

There is evidence that interferon-γ, one of the most potent activators of endogenous macrophages using the JAK-STAT pathway, induces M1-like polarization (18). The pro-inflammatory cytokine IL-6 activates M1-like polarization through the JAK2/STAT pathway, whereas healing factors such as IL-4 and IL-10 promote M2-like polarization (60, 61). In inflammatory immune diseases, multiple biological responses are mediated by the JAK/STAT pathway, with STAT1, STAT3, and STAT6 playing key roles in macrophage polarization (62). Thus, IFN-γ and IL-12 activate JAK, leading to phosphorylation of STAT1 and promoting M1-like polarization; in contrast, IL-4 and IL-13 enhance STAT6 and STAT3 expression and promote M2-like polarization (60, 63). In addition, as an inhibitor of the JAK/STAT pathway, suppressor of cytokine signaling (SOCS) is involved in macrophage polarization, and reduced SOCS1 levels can lead to M1-prone polarization (62). Therefore, the JAK/STAT pathway is a key player in the pathogenesis of autoimmune diseases and a potential target for novel therapeutic strategies.

#### 3.1.3 PI3K/Akt signaling pathway

Activation of PI3K signaling regulates many aspects of cellular activity, including viability, metabolism, motility, and proliferation (64, 65). Akt(also known as PKB) is the most prominent effector of PI3K (66, 67). Evolving evidence suggests that the PI3K/Akt signaling pathway also plays a crucial role in macrophage activation and gene expression (68, 69).

Dysregulated macrophage differentiation has been implicated in infectious and inflammatory diseases (70–72). In infection, Akt kinase contributes differentially to macrophage polarization, with Akt1 ablation leading to M1-like polarization, whereas Akt2 ablation leads to M2-like polarization (72, 73). It has been suggested that Akt1 signaling induced by Staphylococcus aureus is essential in the shift of macrophages from an antimicrobial phenotype (M1) to a functionally inert trait (72).

#### 3.1.4 JNK signaling pathway

The JNK signaling pathway belongs to the mitogen-activated protein kinase (MAPK) superfamily, which is a key regulator of many cellular events, including cell proliferation, transformation, differentiation, and programmed cell death (apoptosis) (74).

It has been suggested that JNK signaling contributes to the development of inflammation and plays an important role in metabolic responses, such as those associated with obesity (75, 76). It has been demonstrated that JNK expression in macrophages may play a key role in the accumulation of adipose tissue macrophages and M1-like polarization in mice (77, 78).

#### 3.1.5 C/EBP family

The C/EBP family is a group of key transcription factors that play a key role in the immune system, particularly in macrophage development, function, and polarization (57). This family includes several members, including C/EBPα, C/EBPβ, and C/EBPγ. It has been shown that C/EBPβ drives M1 formation by regulating proinflammatory cytokines such as IL-6 and TNF-α (79). In contrast, C/EBPα and C/EBPγ play key roles in anti-inflammatory responses and tissue repair, with C/EBPγ associated with M2 polarization, thereby supporting tissue repair and regeneration (80, 81). Macrophage metabolism is also influenced by the C/EBP family, which in turn shapes their functional responses, thereby influencing their polarization state and overall function (82). Thus, by regulating macrophage differentiation, polarization, and metabolism, the C/EBP family profoundly influences the immune response.

#### 3.1.6 Notch signaling pathway

The Notch signaling pathway is an evolutionarily conserved intercellular communication system that mediates juxtacrine signaling through direct membrane contact between adjacent cells and can influence multiple aspects of animal development and tissue renewal processes (83, 84). Generally, aberrant activation of Notch signaling is often associated with macrophage activation and dysfunction (85–87). Relevant evidence indicates that Notch signaling plays a significant role in regulating macrophage function in inflammatory diseases and can exert important effects on macrophage polarization during the progression of atherosclerosis (85) and tumor disease progression (88).

### 3.2 Effects of metabolic reprogramming on macrophage polarization

Various stimuli in the human microenvironment can trigger changes in intracellular metabolic patterns, a process known as metabolic reprogramming. Metabolic reprogramming is a core driver of functional plasticity during macrophage polarization. When disturbed by environmental signals, macrophages retool their functions to execute the immune program. In particular, cellular intrinsic immune metabolites, such as succinate, can act as immunomodulatory signals that affect macrophage activity. Thus, macrophages can acquire new phenotypes and functions through metabolic reprogramming, while also influencing the developmental outcomes of numerous diseases (89, 90).

Numerous studies show that metabolic reprogramming in various diseases profoundly affects macrophage polarization and disease progression. In malignant tumors, the Warburg effect is one of the most well-defined metabolic changes in tumor cells (91). Such alterations in tumor cell metabolism have profound effects on tumor-associated macrophages, which can significantly affect macrophage metabolism and polarization through metabolites such as α-ketoglutarate (α-KG), and ultimately tumor metastasis and progression (92). In addition, in infectious diseases, due to the different types of infection, the body’s glycolysis, amino acid metabolism and other metabolic pathways will also show different forms of changes, so as to provide energy to cells in a more efficient way, and produce a large number of ROS, TNFα, IL-6, IL-4, and other biomarkers closely associated with macrophage polarization toward M1, and exerting regulatory effects on inflammation (93).

Metabolic reprogramming is also important for the expression of inflammation in autoimmune diseases. Studies have shown that the Trp-Kyn-IDO1/AhR axis, which is closely related to amino acid metabolism, significantly affects the function of macrophages in systemic lupus erythematosus. In patients with rheumatoid arthritis, synovial macrophages show reprogramming of multiple amino acid metabolic pathways. Metabolomic studies also show that the amino acid metabolic profile of mice with collagen-induced arthritis is significantly disrupted, and abnormalities in glutamine, arginine, and serine pathways are closely related to the inflammatory state (94). In addition, metabolic reprogramming significantly affects macrophage polarization in diabetes, ischemic stroke, atherosclerosis, and other diseases, and has an important impact on disease prognosis (95–97). In general, metabolic reprogramming plays a prominent role in regulating macrophage polarization. Therefore, therapeutic measures targeting changes in metabolic state may yield new biomarkers and therapeutic strategies for related diseases.

#### 3.2.1 Effect of glucose metabolism on macrophage polarization

In general, M1 is more dependent on glycolysis to support its function, whereas anti-inflammatory M2 relies more on oxidative phosphorylation and fatty acid oxidation to provide the energy necessary for metabolism, highlighting the distinct metabolic requirements of macrophage functional phenotypes during polarization (98, 99). In this process, the influence of different metabolic pathways on macrophages is not singular; each pathway exerts distinct regulatory effects on macrophage polarization from different angles.

Metabolic reprogramming is the core metabolic basis for regulating the polarization and function of macrophages, which mainly includes five pathways: glycolysis, tricarboxylic acid (TCA) cycle, pentose phosphate pathway (PPP), lipid metabolism (including fatty acid oxidation (FAO) and fatty acid synthesis (FAS)), and amino acid metabolism. When pathological responses such as inflammation occur, environmental changes will use the above-mentioned metabolic pathways to synergistically shape macrophage polarization toward M1 and M2, regulate the dynamic shifts in their oxidative metabolism, and play a key role in infection and inflammatory responses (93, 100).

Glycolysis is localized in the cytoplasm, rapidly generates ATP, and provides metabolic intermediates, serving as the core energy source for M1 macrophages. Glycolysis is significantly enhanced in M1 macrophages, with hypoxia-inducible factor-1α (HIF-1α) acting as a key transcription factor that upregulates glycolytic key molecules such as glucose transporter 1, fructose-2,6-bisphosphatase 3, and pyruvate kinase muscle isozyme 2, thereby promoting glucose uptake and glycolytic flux, while supporting the conversion of pyruvate to lactate to sustain continuous glycolytic activity (101, 102). Inhibition of glycolysis directly impairs M1 macrophage functions, including phagocytosis, ROS production, and pro-inflammatory cytokine secretion (93). M2 macrophages exhibit relatively low glycolytic activity and rely more on oxidative phosphorylation and fatty acid oxidation for metabolism. Studies have shown that upregulation of fructose-2,6-bisphosphatase 1 and carbohydrate kinase-like protein can reduce glycolytic flux and maintain the stability of the stability of macrophage polarization toward the M2 phenotype (98).

The TCA cycle, also known as the Krebs cycle, takes place in the mitochondria. The accumulation of its key cycle intermediates and their derivatives in macrophages can generate distinct signaling functions that regulate immune cell activation (103). M2 macrophages possess an intact TCA cycle and efficiently generate energy through oxidative phosphorylation to meet the demands of tissue repair and anti-inflammatory responses (89, 98, 104). In contrast, the TCA cycle in M1 macrophages exhibits two breaks, resulting in abnormal accumulation of citrate and succinate. Succinate can stabilize HIF-1α, further amplifying glycolytic and pro-inflammatory effects, while citrate participates in the synthesis of pro-inflammatory mediators and supports the functional execution of anti-inflammatory macrophages (93).

PPP also serves as a shunt pathway for cellular energy generation. PPP activity is enhanced in M1 macrophages but reduced in M2 macrophages. M1 macrophages predominantly engage in the oxidative phase of the PPP, generating substantial amounts of NADPH, which supports NADPH oxidase-mediated ROS production to eliminate pathogens (93). Furthermore, it has been proposed that Akt and its upstream regulators promote macrophage polarization toward the M1 phenotype, and M1 polarization is also associated with p53-enhanced PPP activity in an Akt- and Specificity Protein 1 (SP-1)-dependent manner. Therefore, p53 may sustain the M1 phenotype by stimulating the PPP through Akt- and SP-1-dependent pathways, and related therapeutic strategies targeting this axis have considerable application potential (105).

Studies have shown that under infectious and inflammatory conditions, pathogens activate macrophages via the NF-κB and Akt/mTOR pathways, driving upregulation of glycolysis and the PPP, as well as reprogramming of the TCA cycle, thereby promoting M1 polarization and initiating anti-infectious inflammation (101, 106). During the resolution phase of inflammation, metabolism shifts toward oxidative phosphorylation and fatty acid oxidation, and macrophages polarize toward the M2 phenotype, mediating the resolution of inflammation and tissue repair (106, 107).

#### 3.2.2 Effect of lipid metabolism on macrophage polarization

Alterations in lipid metabolism also significantly influence inflammatory outcomes and macrophage function. Studies have shown that lipid metabolism can link metabolic reprogramming to inflammatory signaling by regulating pathways such as HIF-1α and NF-κB, as well as receptors such as Cluster of Differentiation 36 (CD36), thereby playing a central regulatory role in infections, tumors, and metabolic diseases (108–111). For example, lipid mediators such as oxylipins, which are endogenous factors produced by both the host and pathogens and are highly regulated and functionally diverse, can serve as key signals mediating the resolution of inflammation. Meanwhile, in the tumor microenvironment, the shift from glycolysis to fatty acid oxidation (FAO) and increased ROS production play critical roles in macrophage polarization (112, 113). Evidence indicates that abnormal lipid metabolism in adipose tissue can induce metabolic inflammation, and plastic macrophages accompanying metabolic abnormalities will rapidly alter their phenotypes and functions; for instance, lipid overload can drive M1 polarization (114, 115). Additionally, macrophages are also important for lipid metabolism, as they can adjust their functions in lipid uptake and turnover in response to different environmental stimuli to prevent lipotoxicity (116). Therefore, lipid metabolism is a key metabolic network regulating macrophage activation, polarization, and inflammatory functions. The dynamic balance among factors such as fatty acid oxidation (FAO), fatty acid synthesis (FAS), and lipid signaling molecules influences macrophage differentiation toward either the M1 or M2 phenotype (99, 117).

The process of macrophage polarization toward the M1 phenotype is profoundly influenced by FAS. Following inflammatory stimulation, macrophage activation leads to the upregulation of FAS-related genes, such as Sterol Regulatory Element-Binding Protein, which catalyze the production of pro-inflammatory precursors and promote macrophage differentiation toward the pro-inflammatory M1 phenotype, thereby enhancing bactericidal and pro-inflammatory functions (101). In contrast, M2 polarized macrophages rely on FAO and oxidative phosphorylation for energy production, and IL-4 and IL-13 can promote fatty acid uptake and oxidation by activating the STAT6 pathway and upregulating activating transcription factor 1 (98). FAO further consolidates M2 polarization by regulating glutaminolysis and histone modifications (98). In addition, Fatty Acid-Binding Protein 5 and Sorting Nexin 10 can also reprogram macrophages by modulating lipid uptake and oxidation, which is conducive to macrophage polarization toward the M2 phenotype (89).

#### 3.2.3 Effect of amino acid metabolism on macrophage polarization

Amino acid metabolism constitutes a key metabolic network that regulates macrophage activation, polarization, and immune functions. Through the differential metabolism of core amino acids such as arginine, glutamine, and tryptophan, the body drives the differentiation toward pro-inflammatory M1 or anti-inflammatory M2 phenotypes, participating in various pathophysiological processes including infection, inflammation, and tissue repair (94, 118). Arginine metabolism serves as a central regulatory axis for macrophage polarization. In M1-polarized macrophages, stimulation by interferon-γ (IFN-γ) and TNF-α induces high expression of inducible nitric oxide synthase (iNOS), which catalyzes the conversion of arginine to nitric oxide (NO) and citrulline, thereby enhancing bactericidal and pro-inflammatory functions while also preventing M1-to-M2 repolarization. In contrast, M2-polarized macrophages, upon IL-4/IL-13 stimulation, upregulate arginase 1 (Arg1), which converts arginine into ornithine and polyamines, thereby promoting tissue repair and immune tolerance while maintaining the M2 phenotype (93, 98, 99, 119).

Glutamine metabolism regulates macrophage polarization through pathways involving α-KG and other intermediates. In M1-polarized macrophages, glutaminolysis generates succinate, which stabilizes HIF-1α, enhances cooperation with cytosolic glycolysis, and reinforces pro-inflammatory signaling. In contrast, M2-polarized macrophages rely on glutamine to produce α-KG, which promotes M2 marker expression through epigenetic reprogramming and supports oxidative phosphorylation for energy production. Glutamine also supports M2 polarization by inhibiting the desuccinylation of pyruvate dehydrogenase. Furthermore, glutamine metabolism modulates the activity of IκB kinase β and inhibits macrophage activation toward M1 through a mechanism dependent on α-KG-Pro hydroxylases, thereby blocking the NF-κB pathway and ultimately leading to M2 polarization (89, 98, 101). These mechanisms indicate that glutamine metabolism plays a significant role in macrophage polarization.

Tryptophan is metabolized by the rate-limiting enzymes indoleamine 2,3-dioxygenase and tryptophan 2,3-dioxygenase to generate kynurenine. Through the kynurenine pathway, cell-autonomous production of NAD+ can modulate macrophage immune functions. In addition, tryptophan metabolism in macrophages can promote immune tolerance by enhancing M2 polarization and depleting extracellular tryptophan, thereby modulating T cell functions (89, 93). Serine promotes glycolysis and mitochondrial metabolism. Serine metabolism contributes to increased glycolytic flux and influences macrophage polarization through multiple mechanisms, including promoting glycolysis, supporting the one-carbon cycle, and regulating epigenetic modifications. Studies have shown that serine synthesis is closely associated with the expression of pro-inflammatory cytokines and iNOS in M1-polarized macrophages, as well as with the proportion of polarization toward the M2 direction (89, 120–122). Furthermore, branched-chain amino acids, glycine, aspartate, and various sulfur-containing amino acids can cooperatively shape macrophage functional phenotypes by regulating mTOR signaling, the NF-κB pathway, one-carbon metabolic flux, mitochondrial metabolism, and redox balance (94, 123).

In addition to the above-mentioned metabolic pathways, creatine can suppress the IFN-γ/JAK/STAT1 axis while promoting the IL-4/STAT6/ARG1 axis, thereby inhibiting M1-like polarization and activating M2-like polarization (124). Furthermore, iron metabolism can influence macrophage polarization by regulating the NF-κB and MAPK pathways (125), while lactate metabolism can promote macrophage polarization toward the M2 phenotype by activating the mTORC2/Akt and cAMP/ICER pathways (126). Therefore, metabolic reprogramming profoundly influences macrophage function and can regulate macrophage polarization through multiple pathways, representing a key direction for exploring clinical therapeutic strategies.

## 4 Metabolic reprogramming and macrophage polarization in PID

### 4.1 Characteristics of metabolic disorders in pelvic inflammatory disease

PID is an inflammatory disease of the upper reproductive tract, and its pathological process involves complex metabolic reprogramming phenomena. A metabolomics study revealed that the plasma metabolic profiles of PID rat models were significantly different from those of the normal control group. The related metabolic biomarkers were primarily associated with disturbances in metabolic pathways, including glycerophospholipid, amino acid, arachidonic acid, and unsaturated fatty acid metabolism. Meanwhile, plasma levels of IL-1β and nitric oxide (NO) in PID rats were significantly elevated, suggesting a close association between pelvic inflammatory responses and metabolic disturbances (127).

Meanwhile, a plasma metabolomics study of fallopian tube infection caused by Chlamydia trachomatis identified significant alterations in metabolic pathways involving amino acid metabolism, glycolysis, and the TCA cycle. These metabolic disturbances are closely associated with persistent pathogen infection and chronic inflammation (128). Notably, pelvic inflammatory disease shares certain metabolic similarities with other pelvic disorders. For example, immunometabolic profiling of adenomyosis revealed that amino acid metabolic pathways, including pyrimidine metabolism and carnitine synthesis, were significantly upregulated in diseased tissues (129). These findings suggest that although different disease types have distinct metabolic profiles, inflammatory diseases in the pelvic region may still share certain features of metabolic reprogramming. However, the connections between the metabolic profiles of related diseases have not yet been clearly confirmed by current research and still require further experimental validation.

### 4.2 Effect of metabolism on macrophage polarization in PID

In the characteristic pathological microenvironment of PID, stimuli such as pathogen infection, local tissue hypoxia, and the enrichment of inflammatory mediators exert profound effects on the metabolic status and polarization of macrophages (**Figure 1**). In general, pathogen-associated molecular patterns can directly regulate macrophage metabolism through Toll-like receptor (TLR) signaling pathways (130, 131). Furthermore, LPS, a major virulence factor of Gram-negative bacteria, plays an important role in the pathogenesis of PID. Studies have shown that LPS induces macrophage polarization toward the M1 phenotype, accompanied by a significant metabolic switch characterized by increased glycolytic flux and suppressed oxidative phosphorylation. This metabolic switch not only provides rapid ATP supply for macrophage polarization toward M1 but also supplies NADPH and ribose through the pentose phosphate pathway, thereby supporting the synthesis of inflammatory mediators and the production of ROS (132, 133).

**Figure 1:**
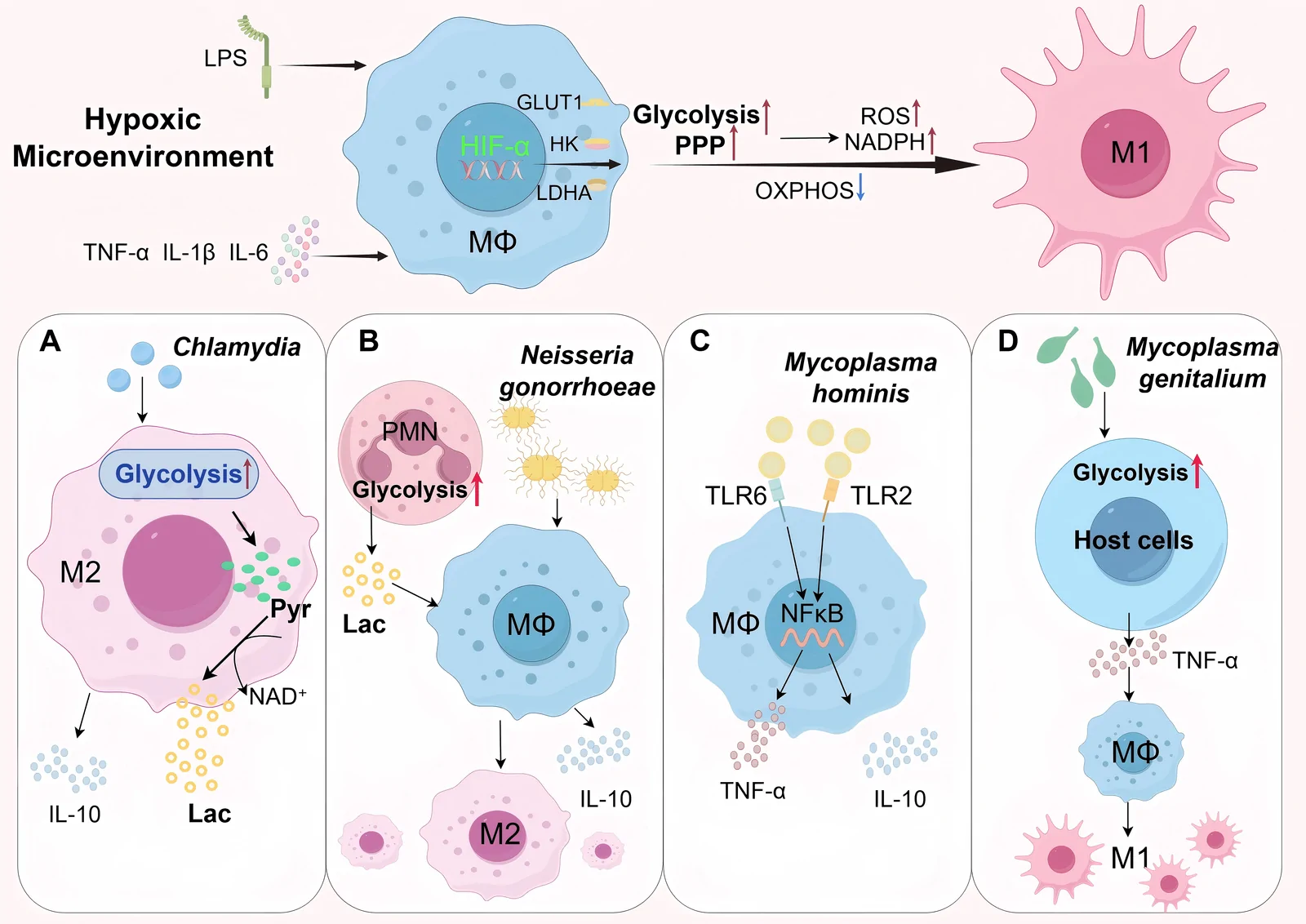
Stimuli such as LPS can elevate levels of proinflammatory factors in the pelvic inflammatory microenvironment, including TNF-α, IL-1β, and IL-6, thereby enhancing glycolysis. More importantly, tissue hypoxia induced by various stimuli stabilizes HIF-1α expression, thereby upregulating glucose transporter 1, hexokinase, and lactate dehydrogenase A to reinforce macrophage glycolysis and the pentose phosphate pathway, generating NADPH and ROS while suppressing oxidative phosphorylation, thus supporting M1 polarization. In addition, Chlamydia can enhance glycolysis in M2 macrophages, promote lactate production, and induce IL-10 generation **(A)**. Neisseria gonorrhoeae can promote M2 phenotype polarization, enhance glycolysis in infiltrating neutrophils in surrounding tissues, leading to lactate accumulation, and simultaneously promote IL-10 production to sustain macrophage polarization toward the M2 phenotype **(B)**. Mycoplasmas can regulate the NF-κB pathway via TLR2/TLR6 signaling, thereby mediating the secretion of IL-10 and TNF-α and affecting macrophage polarization **(C)**. The membrane lipoproteins of Mg can lead to a significant increase in local host cell oxygen consumption, markedly elevate the rate of aerobic glycolysis, and promote the production of the pro-inflammatory cytokine TNF-α, thereby providing a possibility for regulating host macrophage polarization **(D)**. LPS, lipopolysaccharide; MΦ, macrophages; PMN, neutrophil; PPP, pentose phosphate pathway; OXPHOS, oxidative phosphorylation; ROS, reactive oxygen species; NADPH, reduced nicotinamide adenine dinucleotide phosphate; HIF-α, hypoxia-inducible factor-1α; GLUT1, glucose transporter 1; HK, hexokinase; LDHA, lactate dehydrogenase A; Pyr, pyruvate; Lac, lactic acid; NAD+, nicotinamide adenine dinucleotide; NF-κB, nuclear factor-κB; TLR6, Toll-like receptor 6; TLR2, Toll-like receptor 2; TNF-α, tumor necrosis factor-α; IL-1β, interleukin-1β; IL-6, interleukin-6; IL-10, interleukin-10. (Created with www.figdraw.com).

In pelvic inflammatory disease, local tissue hypoxia is common, resulting from vascular injury at the inflammatory site, increased metabolic demand, and reduced oxygen supply (134). The hypoxic environment can further enhance glycolytic metabolism in macrophages by stabilizing HIF-1α, which serves as the core transcription factor regulating aerobic glycolysis and can directly upregulate the expression of glycolytic enzymes such as glucose transporter 1, hexokinase (HK), and lactate dehydrogenase A (135, 136). Studies have shown that HIF-1α expression is significantly elevated in uterine tissues in animal models of pelvic inflammatory disease and is positively correlated with the severity of inflammation (137, 138).

Therefore, the positive feedback loop of the hypoxia/HIF-1α/glycolysis axis may be a key mechanism exacerbating macrophage polarization toward M1 during the pathological progression of PID, leading to further tissue damage and aggravated inflammation.

Hexokinase-II (HK-II), a key rate-limiting enzyme of the glycolytic pathway, holds significant potential in regulating the immunometabolism of macrophages associated with PID (139). Studies have found that in a mouse model of LPS-induced pelvic inflammatory disease, inhibition of HK-II-mediated glycolysis effectively alleviated uterine tissue inflammation, reduced infiltration of M1 macrophages (CD86^+^), and concurrently upregulated the expression of the M2 marker CD206 (132, 140).

Furthermore, inflammatory cytokines in pelvic inflammatory disease can also reciprocally affect the metabolic status of macrophages. Pro-inflammatory cytokines such as TNF-α, IL-1β, and IL-6 are significantly elevated in the serum and local tissues of patients with pelvic inflammatory disease (141). These factors can further enhance the expression of glycolysis-related genes, thereby providing the possibility of a positive feedback loop between inflammation and metabolism during the pathological progression of PID.4.3 Effects of pathogens on metabolism and macrophage polarization.

Different pathogens play important roles in the pathogenesis of PID, and they all have the potential to regulate macrophage metabolism through their own distinct mechanisms. Chlamydia trachomatis is one of the most common pathogens of PID, with approximately 50–80% of chlamydial infections presenting with subclinical manifestations, and the resulting chronic inflammation and sequelae impose the most severe burden (142). Metabolomics studies have revealed that fallopian tube cells undergo significant metabolic reprogramming following chlamydial infection, thereby providing the ATP and biosynthetic precursors required for intracellular chlamydial growth (128).

Meanwhile, chlamydial infection induces macrophages to increase glucose uptake and lactate production. This metabolic reprogramming not only represents a host cellular response to infection but is also exploited by the pathogen to acquire carbon sources required for its growth. Interestingly, however, although chlamydial infection drives macrophages to exhibit M1-like metabolic features such as enhanced glycolysis, these metabolic alterations predominantly occur in M2 macrophages (**Figure 1A**). This suggests that the glycolytic changes induced by Chlamydia are more directly involved in sustaining pathogen survival, while also indicating that Chlamydia may have the ability to regulate lactate accumulation in neighboring cells and induce cytokine production to modulate macrophage polarization (128, 143).

Neisseria gonorrhoeae is another important pathogen of PID, and its infection is characterized by robust neutrophil infiltration (144). Evidence indicates that N. Gonorrhoeae can promote macrophage polarization toward the M2 phenotype. At the metabolic level, during N. Gonorrhoeae infection, glycolysis in neutrophils is significantly enhanced, exhibiting Warburg-like metabolic changes that result in substantial lactate production and accumulation. This creates conditions conducive to M2 polarization and promotes IL-10 production, which further sustains macrophages’ propensity toward M2 polarization (**Figure 1B**) and facilitates immune evasion (145–147). But research into how Neisseria gonorrhoeae influences the pathological progression of PID through metabolic regulation of macrophage polarization remains limited, and further experiments are needed to clarify the specific targets.

Ureaplasma urealyticum and Mycoplasma hominis are opportunistic pathogens of PID that can cause disease under specific conditions (148, 149). Mycoplasmas can influence pathways such as NF-κB through systems like TLR2/TLR6, mediating the secretion of cytokines including IL-10 and TNF-α (**Figure 1C**), and similarly have the potential to affect macrophage function and polarization status (150, 151).

Mycoplasma genitalium (Mg) is one of the important pathogens of PID. Similar to Chlamydia trachomatis, its infection often presents with subclinical manifestations, but its pathogenicity is closely associated with its unique carbon metabolic pathway (152). Mg possesses a distinctive glycerol metabolic pathway, which may be involved in various pathophysiological and pathogenic processes. Studies have shown that during glycerol metabolism, Mg can produce large amounts of hydrogen peroxide, which can directly damage host cells and cause oxidative stress injury (153). Meanwhile, the membrane lipoproteins of Mg can lead to a significant increase in local host cell oxygen consumption, markedly elevate the rate of aerobic glycolysis, and promote the production of the pro-inflammatory cytokine TNF-α (154). Therefore, Mg not only promotes the generation of metabolically toxic products, but also enhances glycolysis and promotes the production of TNF-α, a cytokine that activates macrophage polarization toward M1. This provides a possibility for Mg to regulate host macrophage polarization through metabolic modulation (**Figure 1D**).

In summary, different pathogens play important roles in the pathogenesis of PID, and they may regulate macrophage metabolism through their own distinct mechanisms to influence disease progression. However, the etiology of PID is essentially polymicrobial in nature, and the microbial ecosystem of the lower genital tract is not dominated by a single pathogen, but rather constitutes a complex network of multiple aerobic bacteria, anaerobic bacteria, and sexually transmitted pathogens. In addition, similar to studies on the mechanisms by which the PID microenvironment metabolically regulates macrophage polarization, research on the mechanisms by which different pathogens regulate macrophage polarization through metabolism is currently mostly based on indirect evidence. The metabolic mechanisms of some pathogens have not yet been clearly confirmed and still require further experimental validation (**Table 1**).

**Table 1:** Evidence level summary of mechanisms related to metabolic reprogramming and macrophage polarization in PID.

Mechanism	Direct evidence in PID	Indirect evidence	Model used	Functional consequence	Confidence level
Metabolic disorders in PID	Limited (127)	adenomyosis (129)	Animal/human tissue samples	Metabolic disorders in the PID microenvironment	Medium
Hypoxia/HIF-1α/glycolysis/M1	Mainly endometritis (137, 138)	Hypoxia-induced (135)/LPS (136)	Animal/*in vitro*	Tissue damage/Inflammation	Medium
HK-II/glycolysis/M1	Limited (132, 140)	LPS (133)	Animal/*in vitro*	Tissue damage/Inflammation	Medium
Pro-inflammatory cytokines/glycolysis/M1	Limited (132, 140)	LPS (133)	Animal/*in vitro*	Tissue damage/Inflammation	Medium
Glycolysis/M2	Indirect	Chlamydia trachomatis/human immortalized fallopian tube cells (128, 143)	Infection/*in vitro*	Maintains pathogen survival	Low-medium
Lactate/M2	Indirect	Neisseria gonorrhoeae/neutrophils (145–147)	Infection/*in vitro*	Immune evasion	Low-medium
TLR/NF-κB/cytokines/macrophage activation	Indirect	Mycoplasma hominis/macrophages (150, 151)	Infection/*in vitro*	Inflammation	Low-medium
Glycolysis/TNF-α/M1	Indirect	Mycoplasma genitalium/human urethral epithelial cell line	Infection/*in vitro*	Inflammation	Low

Confidence level: Medium — Supported by direct PID-related studies, or indirect evidence is substantial and derived from high-quality models with reasonable inference; Low-medium — Supported by indirect evidence, but derived from non-PID models, with some rationale for inference but requiring validation; Low — Evidence mainly from other disease models or *in vitro* experiments, with insufficient PID-specific verification; Very low — Based only on theoretical speculation or epidemiological associations, lacking direct mechanistic studies.

### 4.3 Application prospects in the treatment of PID

Currently, an in-depth understanding of macrophage metabolic reprogramming provides a theoretical basis for developing novel therapeutic strategies, and interventions targeting key metabolic nodes have shown therapeutic potential (155).

2-Deoxy-D-glucose (2-DG), as an HK-II inhibitor, can regulate macrophage polarization by inhibiting glycolysis (156). The animal experimental study by Pandey S et al. found that 2-DG intervention in the LPS-induced inflammatory model mice effectively reduced oxidative stress responses and slowed the progression of inflammation. This provides further possibilities for exploring therapeutic strategies for PID, which is also an inflammatory disease (157). In addition, studies have confirmed that the use of HIF-1α activators in animal models and cell models can increase glucose uptake, promote glycolysis in macrophages, and facilitate M1 polarization. Meanwhile, the PPARγ agonist (pioglitazone), the AMPK activator (metformin), and the rational application of nanomaterials have been shown under experimental conditions to modulate metabolic reprogramming, polarizing toward the M2 phenotype and thereby regulating immune system functions (155). Therefore, therapeutic strategies targeting metabolic reprogramming to regulate macrophage polarization may be relevant to various inflammatory diseases, and their application in PID also warrants further exploration. However, current research remains largely confined to animal and cell-based experiments, so the specific mechanisms and efficacy still require further in-depth investigation and validation through relevant experiments.

In addition to the aforementioned pharmacological strategies that directly target macrophage metabolism to regulate polarization, the emerging neuro-immune regulation has also provided a novel perspective for the adjunctive therapy of PID (158). The vagus nerve, as the primary pathway of the parasympathetic nervous system, can release acetylcholine, reduce the release of pro-inflammatory cytokines during inflammation, and modulate macrophage function (159). Recent studies have further demonstrated that transcutaneous auricular vagus nerve stimulation (ta-VNS) can regulate global lipid and glucose metabolic pathways in humans (160), thereby attenuating the intensity of inflammatory responses. Meanwhile, VNS can also regulate macrophage polarization from the pro-inflammatory M1 phenotype to the anti-inflammatory M2 phenotype through the α7nAChR signaling pathway (161). Therefore, VNS can modulate immune system function by targeting metabolic reprogramming and macrophage polarization, alleviate systemic inflammatory responses, and exert therapeutic effects on peripheral system diseases within the vagal innervation territory.

For instance, VNS has already demonstrated favorable clinical safety and efficacy in diseases such as rheumatoid arthritis (161). Given that the pelvis is a region with dense visceral innervation, its inflammatory processes are closely associated with the nervous system, which provides further possibilities for the development of adjunctive therapeutic strategies for PID targeting metabolic reprogramming and macrophage polarization. However, current research on the application of VNS in PID remains relatively limited. Future studies are needed to further verify the specific effects of VNS on macrophage function in PID models and to explore its synergistic effects with existing anti-infective therapies.

Macrophage metabolic reprogramming in PID is closely associated with disease onset and progression. Pathogen infection, a hypoxic microenvironment, and inflammatory cytokines collectively shape macrophage metabolic states, while metabolic changes in turn drive macrophages to polarize toward specific phenotypes. A deeper understanding of this relationship not only helps elucidate the pathogenesis of PID but also provides important directions for the future development of novel adjunctive therapeutic strategies for PID.

## 5 Interaction between endometrial cells and macrophages in PID

In the pathological progression of PID, macrophages do not function in isolation; rather, their regulatory effects on inflammation are accomplished through the formation of a complex bidirectional communication network with cellular components in the endometrial microenvironment. This intercellular crosstalk not only determines the direction of pelvic inflammatory progression but also plays a critical role in tissue injury, repair, and fibrosis formation (**Figure 2**). However, although there is currently a certain foundation of research on the communication between endometrial cells and macrophages, studies specifically related to PID remain relatively limited, and direct evidence is still insufficient, which urgently needs to be further supplemented (**Table 2**).

**Figure 2:**
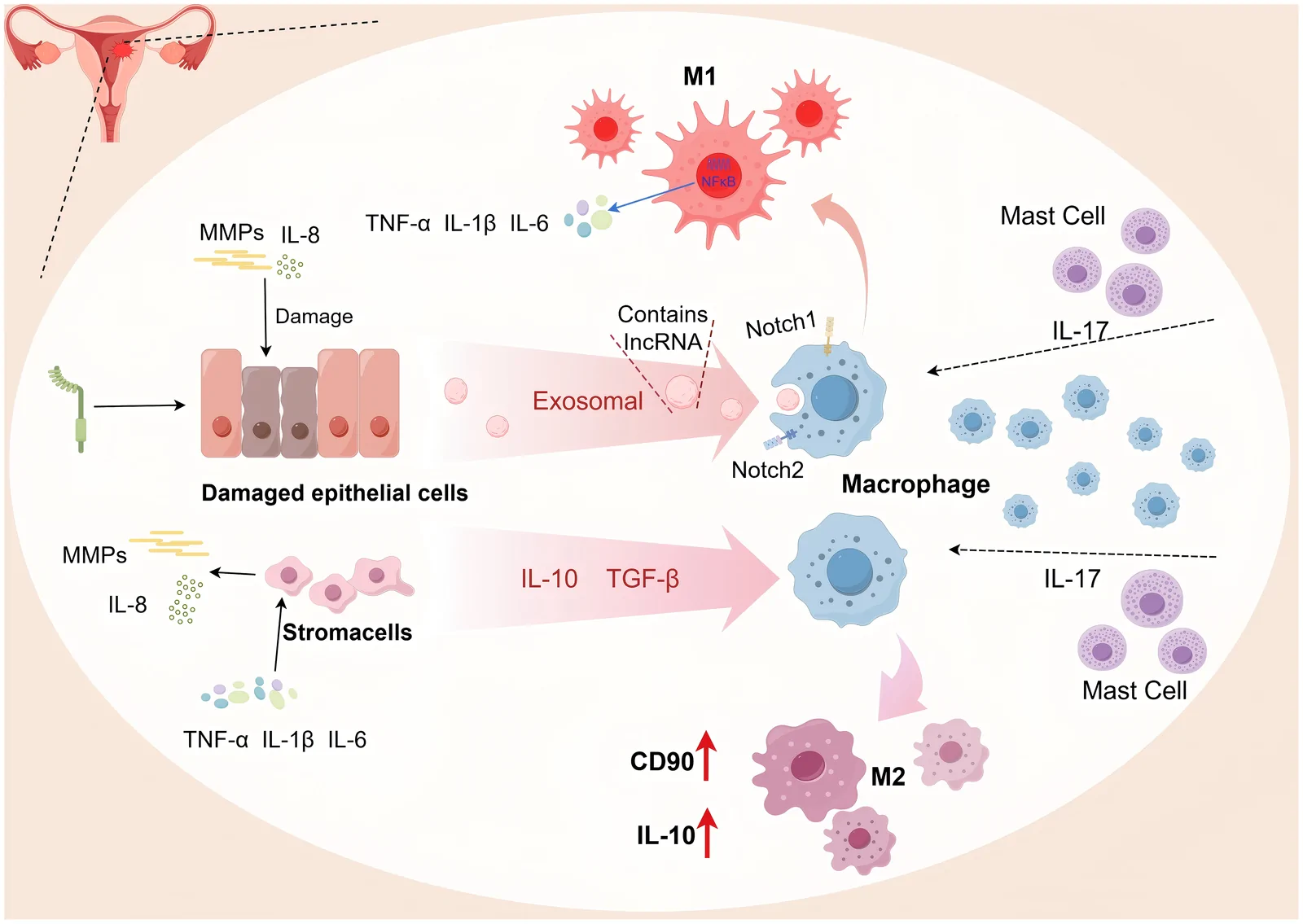
In the pelvic inflammatory microenvironment, IL-17 cytokines secreted by endometrial cells recruit macrophages. Damaged endometrial epithelial cells generate exosomes containing lncRNAs, which are internalized by macrophages, activate the Notch2 pathway to induce M1 polarization, and regulate the Notch1 pathway to mediate the NF-κB pathway in M1 macrophages, enhancing the production of TNF-α, IL-6, and IL-1β. This exacerbates tissue damage in PID and stimulates stromal cells to produce large amounts of the chemokine IL-8 and matrix metalloproteinases. Meanwhile, activated stromal cells induce macrophage polarization toward the M2 phenotype by secreting immunomodulatory mediators such as IL-10 and TGF-β, causing epithelial cell damage. M2 polarization and the increased expression of the endometrial mesenchymal stem cell marker CD90 can occur synchronously and promote the secretion of the anti-inflammatory cytokine IL-10, facilitating normal repair processes. Meanwhile, the contact area between mast cells and monocytes in the inflammatory microenvironment is increased, which may promote monocyte polarization toward M1 or M2 through juxtacrine and paracrine effects. NF-κB, nuclear factor-κB; IL-17, interleukin-17; TNF-α, tumor necrosis factor-α; IL-1β, interleukin-1β; IL-6, interleukin-6; IL-8, interleukin-8; MMPs, matrix metalloproteinases; TGF-β, transforming growth factor-β; IL-10, interleukin-10; CD90, a marker of endometrial mesenchymal stem cells. (Created with www.figdraw.com).

**Table 2:** Evidence level summary of mechanisms related to macrophage-endometrial cell interactions in PID.

Mechanism	Direct evidence in PID	Indirect evidence	Model used	Functional consequence	Confidence level
M1/cytokines/end ometrial stromal cells/IL-8 and matrix metalloproteinases	Indirect	Endometriosis(162)	Primary cells *in vitro*	Tissue damage/Inflammation	Low-medium
M2/endometrial epithelial/stem-pro genitor cell restoration	Indirect	Intrauterine adhesions(163)	Animal/*in vitro*	Tissue repair	Low-medium
M2/CD90/IL-10	Indirect	Intrauterineadhesions(164)	Animal/*in vitro*	Tissue repair	Low-medium
Endometrial exosomes/Notch2/M1	Mainly endometritis (165)	Not always PID-specific	Animal(bovine)/*in vitro*	Tissue damage/Inflammation	Medium
Endometrial exosomes/Notch1 and NF-κB/cytokines	Mainly endometritis (166)	Not always PID-specific	Animal(bovine)/*in vitro*	Tissue damage/Inflammation	Medium
Endometrial stromal cells/IL-10, TGF-β/M2	Indirect	Complications of polypropylene mesh implantation/endometriosis (167, 168)	Human tissue samples/animal	Tissue repair/Tissue fibrosis	Medium
Mast cells/juxtacrine and paracrine/macroph age polarization	Mainly endometritis (169)	Not always PID-specific	Human tissue samples	Inflamma tion/Tissue fibrosis	Low-medium

Confidence level: Medium — Supported by direct PID-related studies, or indirect evidence is substantial and derived from high-quality models with reasonable inference; Low-medium — Supported by indirect evidence, but derived from non-PID models, with some rationale for inference but requiring validation; Low — Evidence mainly from other disease models or *in vitro* experiments, with insufficient PID-specific verification; Very low — Based only on theoretical speculation or epidemiological associations, lacking direct mechanistic studies.

### 5.1 Regulation of endometrial cells by macrophages in PID

During the progression of PID, activated macrophages can directly influence endometrial cell function by secreting inflammatory mediators. Studies have shown that in PID, large amounts of TNF-α, IL-1β, and IL-6 produced by M1 macrophages can stimulate the activation of endometrial stromal cells, inducing them to produce large quantities of chemokines (such as IL-8) and matrix metalloproteinases, which act on endometrial epithelial cells, disrupt their barrier integrity, and exacerbate the inflammatory response (162).

Meanwhile, studies have confirmed that M2 polarization is positively correlated with the recovery of endometrial epithelial cells and stem/progenitor cells (163).

During the repair process of PID, M2 polarization and the increased expression of the endometrial mesenchymal stem cell marker CD90 can occur synchronously and promote the secretion of the anti-inflammatory cytokine IL-10, thereby reducing endometrial inflammation and facilitating normal repair processes. This suggests that M2 may play a critical role in the repair of PID and the development of sequelae (164).

### 5.2 Regulation of macrophages by endometrial cells in PID

During the progression of PID, damaged or infected endometrial epithelial cells and stromal cells can also actively regulate the recruitment and polarization of macrophages. Studies have shown that upon pathogen infection or inflammatory stimulation, damaged endometrial cells can recruit macrophages through autophagy and the secretion of various cytokines (13, 14).

More importantly, under the pathological conditions of PID, extracellular vesicles derived from endometrial cells play a significant role in macrophage polarization. Studies have demonstrated that in experimental models of pelvic inflammatory disease, exosomes derived from LPS-damaged endometrial epithelial cells can be internalized by macrophages, and the lncRNAs within these exosomes can induce macrophage polarization toward the proinflammatory M1 phenotype by regulating specific signaling pathways, such as the Notch2 pathway (165). Furthermore, endometrial epithelial cells regulate the expression of specific miRNAs in exosomes, thereby modulating Notch1 axis expression and NF-κB phosphorylation levels, enhancing the polarization process of macrophages toward M1, and further aggravating tissue damage in PID (166).

Furthermore, under chronic inflammatory conditions in the pelvic cavity, activated endometrial stromal cells can also induce macrophage polarization toward the M2 phenotype by secreting immunomodulatory mediators such as IL-10 and TGF-β (167, 168). Meanwhile, studies have found that the inflammatory microenvironment of the endometrium can activate mast cells near endometrial cells, increasing the contact area between mast cells and monocytes, which may promote monocyte polarization toward M1 or M2 through juxtacrine and paracrine effects (169).

In summary, during the onset and progression of PID, the bidirectional regulation between macrophages and endometrial cells can form a self-sustaining cycle. This intercellular communication network, unique to PID, is a key mechanism in determining the outcome of PID. Disrupting the aberrant communication between macrophages and endometrial cells may provide potential targets for the development of novel adjunctive therapeutic strategies.

## 6 Conclusion

Macrophage polarization regulated by metabolic reprogramming is critical in the development of various immune and infectious diseases, and its important role has been widely confirmed. However, its relevance in PID has not yet been fully explored. The pathological progression of PID is profoundly dependent on macrophage polarization; nevertheless, within the PID microenvironment, macrophage polarization is not an isolated event but is collectively determined by a complex regulatory network comprising multiple signaling pathways and metabolic reprogramming. More critically, macrophages are not only sensitive to the metabolically altered pelvic microenvironment, but they also establish close bidirectional communication with endometrial cells, forming a tightly interconnected network.

Current studies have preliminarily revealed the potential associations among metabolic reprogramming, macrophage polarization, and intercellular communication in PID, and these findings provide a novel conceptual framework for understanding the immunopathological mechanisms of this disease. However, most of the existing knowledge is still based on indirect inferences, and direct evidence derived from PID patients or PID-specific animal models in this field remains quite limited. At the mechanistic level, for instance, the functional validation of the HIF-1α/glycolysis/M1 polarization axis and pathogen-specific metabolic regulation in PID is still insufficient. Although these indirect pieces of evidence offer clues for hypothesis generation, they cannot replace the direct characterization of macrophage metabolic status within the specific pathological context of PID itself.

From the perspective of therapeutic translation, strategies targeting metabolic nodes such as HK-II, HIF-1α, or PPARγ have shown certain potential, and neuroimmune interventions such as vagus nerve stimulation also provide new directions for future therapy. However, these studies are still in the experimental exploration stage. Translating these findings into adjunctive treatment regimens applicable to PID patients still requires overcoming numerous obstacles, including the synergistic optimization of immunometabolic modulation and antimicrobial therapy.

In summary, metabolic reprogramming-driven aberrant macrophage polarization and intercellular communication may represent a regulatory dimension worthy of attention in the pathological progression of PID. However, the exploration of this mechanism urgently requires validation through more direct interventional studies, including multi-omics approaches such as metabolomics and single-cell transcriptomics, targeting PID patients and their specific pathogen spectra, so as to lay the foundation for the development of truly clinically valuable precision intervention strategies.
